# Role of TRPC1 channels in pressure-mediated activation of airway remodeling

**DOI:** 10.1186/s12931-019-1050-x

**Published:** 2019-05-15

**Authors:** Na Li, Ye He, Gang Yang, Qian Yu, Minchao Li

**Affiliations:** 1 Department of Respiratory Medicine, The Second Affiliated Hospital of Chongqing Medical University, Chongqing, 400010 People’s Republic of China; 2 Department of Geriatrics, Sichuan Provincial People’s Hospital, Sichuan Academy of Medical Science, Chengdu, Sichuan Province 610072 People’s Republic of China; 3 Department of Neurosurgery, The First Affiliated Hospital of Chongqing Medical University, Chongqing, 400010 People’s Republic of China

**Keywords:** TRPC1 channel, Ca^2+^, Airway remodeling, Asthma

## Abstract

**Background:**

Bronchoconstriction and cough, a characteristic of the asthmatic response, leads to development of compressive stresses in the airway wall. We hypothesized that progressively pathological high mechanical stress could act on mechanosensitive cation channels, such as transient receptor potential channel 1 (TRPC1) and then contributes to airway remodeling.

**Methods:**

We imitate the pathological airway pressure in vitro using cyclic stretch at 10 and 15% elongation. Ca^2+^ imaging was applied to measure the activity of TRPC1 after bronchial epithelial cells exposed to cyclic stretch for 0, 0.5, 1, 1.5, 2, 2.5 h. To further clarify the function of channnel TRPC1 in the process of mechano-transduction in airway remodeling, the experiment in vivo was implemented. The TRPC1 siRNA and budesonide were applied separately to asthmatic models. The morphological changes were measured by HE and Massion method. The expression levels of TRPC1 were evaluated by real-time PCR, western blot and immunohistochemistry. The protein expression level of IL-13, TGF-β_1_ and MMP-9 in BALF were measured by ELISA.

**Results:**

The result showed that cyclic stretch for 15% elongation at 1.5 h could maximize the activity of TRPC1 channel. This influx in Ca^2+^ was blocked by TRPC1 siRNA. Higher TRPC1 expression was observed in the bronchial epithelial layer of ovalbumin induced asthmatic models. The knockdown of TRPC1 with TRPC1 siRNA was associated with a hampered airway remodeling process, such as decreased bronchial wall thickness and smooth muscle hypertrophy/hyperplasia, a decreased ECM deposition area and inflammation infiltration around airway wall. Meantime, expression of IL-13, TGF-β_1_ and MMP-9 in OVA+TRPC1 siRNA also showed reduced level. TRPC1 intervention treatment showed similar anti-remodeling therapeutic effect with budesonide.

**Conclusions:**

These results demonstrate that most TRPC1 channels expressed in bronchial epithelial cells mediate the mechanotransduction mechanism. TRPC1 inducing abnormal Ca^2+^ signal mediates receptor-stimulated and mechanical stimulus-induced airway remodeling. The inhibition of TRPC1 channel could produce similar therapeutic effect as glucocortisteroid to curb the development of asthmatic airway remodeling.

## Background

As a dynamic organ, cells and intracellular and extracellular matrix (ECM) in the pulmonary are under the influence of transpulmonary pressure and the incessant mechanical stresses during breathing [1], and excessive mechanical stress to the bronchial epithelial cells is one of the most common characters of chronic inflammatory airway diseases, such as asthma [2], as a result of exacerbation triggered by inhalation of allergens or viral infections [3, 4]. Exacerbation that accompanies bronchoconstriction and chronic cough leads to the pathological upregulation of small and medium airway pressure [2, 5–7].

Recent studies have shown that the upregulated airway pressure acts as a mechanical stimulus that produces chronic sustained and positive pressure in pulmonary tissue [2, 5–7]. Some researchers proposed that the bronchoconstriction that accompanies mechanical stress contributes to airway remodeling, in the same way that mechanical stimuli lead to remodeling of musculoskeletal [8, 9] and cardiovascular [10] tissues. For example, short duration episodes of mechanical stress, are sufficient to increase goblet cell number and mucin5AC (MUC5AC) protein expression in bronchial epithelial cells [11, 12]. Chronic cyclical stretch applied to tracheal epithelial cells, human airway smooth muscle cells upregulates the transforming growth factor (TGF-β_1_) in pressure-dependent manner [7, 13, 14], which could result in ECM deposition of human vascular smooth muscle cells [15] and airway smooth muscle cells [15, 16]. Mechanical stress on human airway epithelial cells elicits a matrix remodeling response in unstressed, cocultured pulmonary fibroblasts through soluble signals [17]. However, molecular mechanisms of mechanotransduction that link physical forces to intracellular signalling pathways remain elusive.

The transient receptor potential channel 1 (TRPC1) was firstly cloned among TRPs [14] and exhibited high mechanosensitivity in vertebrates [18]. Additionally, Ca^2+^ influx evoked by the activation of TRPC1 can regulate and promote cell proliferation, differentiation, secretion and migration [19–21]. The regulatory mechanism of intracellular Ca^2+^ has the ability to modulate the expression of many chemokines and cytokines, such as interleukin-13 (IL-13), TGF-β_1_, collagen deposition and matrix metalloproteinase-9 (MMP-9) [22–24]. These above-mentioned activities imply an intimate association between TRPC1-Ca^2+^ influx and the development of airway remodeling. However, the exact significance of the expression of TRPC1 in pulmonary tissue is poorly understood. Previous studies in this field have primarily been limited to the lack of specific TRPC1 antibody [25]. Therefore, we hypothesized that TRPC1 may play important roles in the process of mechanotransduction in airway remodeling. The early administration of budesonide may be able to significantly suppress airway inflammation, which is an important factor that result in airway remodeling. Although the clinical use of budesonide has the potential to reverse airway remodeling, the underlying mechanism remains to be explored.

In this study, based on character of TRPC1-coupled calcium influx and Flexcell FX-4000 Tension System, we imitated the pathological airway mechanical pressure. We identify TRPC1 channel as a mechanical channel, which could be maximized at 1.5 h, 15% elongation. In vivo, most TRPC1 protein expanded around bronchial epithelial layer in asthmatic models. Using knockdown followed by siRNA intervention, results showed lower expression levels of bronchial wall thickness, abnormal airway smooth muscle hypertrophy and hyperplasia, collagen deposition, inflammation infiltration around airway and inflammatory chemokines (IL-13, TGF-β_1_ and MMP-9). These pathological changes showed similarly with budesonide intervention. Take together, our findings suggest that TRPC1 may trigger and aid in the development of asthmatic airway remodeling. Down-regulation of TRPC1 has similar clinic therapeutic effect with budesonide to target asthmatic airway remodeling.

## Methods

### Cell culture

Sixteen human bronchial epithelial cells (16HBE) (ATCC Rockville, USA) were cultured in RPMI 1640 (Gibco, USA) medium supplemented with 10% fetal bovine serum (Gibco, USA), 100 units/ml penicillin (Boster, China) and 100μg/ml streptomycin (Boster, China) and incubated at 37 °C in a humidified water-jacketed incubator containing 95% air and 5% CO_2_ during the subsequent experiments.

### Stable transfection of TRPC1 siRNA

The 16HBE cells were plated into 6-well plates at 200,000 cells per well in RPMI 1640 medium containing 10% fetal bovine serum and incubated overnight. 16HBE cells were then cultured in serum-free medium before being divided into 3 groups: a control group, a TRPC1siRNA group and a NC siRNA group. At 6 h after transfection, the cells were fed with medium and incubated for another 48 h. Immunofluorescence and western blot analysis were then performed to determine the efficiency of the TRPC1 knockdown.

### Mechanical stretch system

In the in vitro studies of bronchial epithelial cells, the cells were stimulated using cyclic stretch to imitate the pathological conditions associated with airway mechanical stress. Cells were first seeded into Flexcell biaxial 6-well culture plates (coated with type I collagen, BioFlex-I; Flexcell international Corp., USA) at a density of 400,000 cells per well in medium for 24 h. Then, firstly, the cells were cultured in serum-free medium and divided into 2 groups: a control group, a stretch group. In the stretch process, as previous described in our report [26]. Cells were subjected to a sinusoidal cyclic stretch of 10 and 15% elongation at 1 Hz using a Flexcell FX-4000 Tension System (Flexcell International Corp., USA). We used Ca^2+^ imaging to measure the activation of TRPC1 in 16HBE cells after the cells were exposed to cyclic stretch for 0, 0.5, 1, 1.5, 2, 2.5 h. Secondly, cells were divided into 4 groups: a control group, a stretch group, a stretch+TRPC1 siRNA group, a stretch+NC siRNA group. The cells were subjected to a sinusoidal cyclic stretch of 15% elongation.

### Immunofluorescence

Cells were seeded into 24-well plates a density of 100,000 cells per well and cultured in RPMI1640medium overnight. The cells were washed by PBS, fixed with 4% paraformaldehyde and permeabilized using 0.3% Triton X-100. Rabbit anti-TRPC1 polyclonal antibodies (Abcam, USA) were diluted 1:100. Then, all of the culture dishes were incubated with FITC-labelled goat anti-rabbit antibodies (1:100, Cell Signaling Technology, USA) and stained with DAPI (10 mg/ml, Abcam, USA). Fluorescent labeling was analyzed using fluorescent upright microscope (BX51, Olympus). Fluorescence intensities were evaluated using Image-pro Plus software (Media Cybernetics). Results are shown as fold control of fluorescence intensity.

### Ca^2+^ imaging

After the cells were stretched, they were seeded into confocal dishes at a density of 1,000,000 cells per dish. After the cells were washed 3 times with PBS, they were incubated with PBS containing 3 mmol/L Flou3-AM (Beyotime, China) for 45 min at 37 °C to load the Ca^2+^ indicator. After the cells were washed three times using modified Krebs-Ringer buffer, they were finally incubated at 37 °C for an additional 30 min. Images were obtained and analysed using a confocal laser-scanning microscope system (Leica TCSSP2). The quantification tools available in the confocal microscope software were used to evaluate fluorescence intensity. Results are shown as fold control.

### Animal models with airway remodeling

Six to eight weeks of male guinea pigs weighting 130 g to 170 g were acquired (SCXK (yu) 2007–0001, experimental Animal Centre, Chongqing Medical University). Groups of 34 guinea pigs were housed in plastic cages in a standard animal care facility (Experimental Animal Centre, Chongqing Medical University) with free access to food and water.

Chronic asthmatic models were constructed as described in previous studies [27]. Two groups (*A* and *B*) were included in this study. The *A* group contained nine animals that were randomly distributed into control group, TRPC1 siRNA group and NC siRNA group to test the interfering effect of siRNA. The guinea pigs in the *B* group were randomly divided into five groups: control group (*n* = 5); ovalbumin exposure group (OVA group, *n* = 5); ovalbumin exposure + NC siRNA group (OVA+NC siRNA group, *n* = 5); ovalbumin exposure + TRPC1 siRNA group (OVA+TRPC1 siRNA group, *n* = 5); and ovalbumin exposure + budesonide group (OVA+budesonide group, *n* = 5). Ovalbumin exposure (grade III, Sigma USA) was performed through an intraperitoneal injection (ip) with 10 g/L ovalbumin solution and 10 g/L aluminium hydroxide (Kelong Chemical, Chengdu, China) in 0.9% saline (1 mL) on days 0 and 7. Starting on day 14, 1% ovalbumin solution (2 mL) was administered every other day for 6 weeks by air atomizer (Jiangsu Yuyue Medical, 403C). Prior to ovalbumin exposure, animals in TRPC1 siRNA group were pretreated with TRPC1 siRNA (Shanghai GenePharmaCo., Ltd., China) once a week after ip of narcosis, apply 2.5 ml/kg body weight of ready-to-use mixture (1.0 mg/ml medetomedin, 5 mg/ml midazolam, 0.05 mg/ml fentanyl). For gene silencing [28], 60 pmol of siRNA and 5 μL of liposome (Invitrogen, Carlsbad, CA) were dissolved in 100 μL of opti-MEM (Xinfan, Shanghai, China). siRNA was administered via the trachea using a MicroSprayer aerosolizer (Penn Century, Huage Biology Chemical Co., Shanghai, China). Ovalbumin was then administered 2 h after TRPC1 siRNA treatment. The animals in the budesonide group was treated with budesonide solution (0.5 mg/kg, astraZeneca Pty Ltd., Australia) prior to ovalbumin exposure, and 0.9% saline solution was given to the control group. *A* group was sacrificed after 3 days and *B* group after 8 weeks. All of the animals were euthanized by narcosis and handled in accordance with the guidelines of the principles of the Laboratory Animal Care and Use committee [29].

### Surgical procedure

After the final exposure to ovalbumin, all of the animals were anesthetized. A 20-gauge needle was cannulated into the left trachea, and bronchialveolar liquid (BALF) was obtained and kept in PBS. The right pulmonary was maintained for the following analysis.

### Analysis of BALF cells

BALF was performed on the left lung. The BALF cells were washed twice with 1% FBS and then stained with Wright-Giemsa solution [30, 31]. The number of eosinophils was calculated using a haemocytometer chamber.

### Light microscopy and morphometry inspections

The right pulmonary sections were stained with haematoxylin-eosin (HE) and Masson’s stain. The morphological changes were measured using Image Pro software. The inflammatory infiltration score was calculated as follows: no positive cells = 0 points, 1–10% positive cells = 1 point, 11–50% = 2 points, and 51–100% = 3 points [31]. The thickness of the smooth muscle layer (the transverse diameter) = smooth muscle layer thickness/length (μm) of basement membrane [32]. The mean thickness of the bronchial epithelial layer = bronchial epithelial layer thickness/length (μm) of basement membrane [32, 33]. The mean collagen thickness = collagen deposition area / length (μm) of basement membrane [33, 34]. Morphometry was implemented by individuals blinded to the protocol design. Right pulmonary tissue was used for the morphological analysis via a minimum of 10 fields throughout the upper and lower pulmonary.

### Real-time PCR analysis, immunohistochemistry, western blot and ELISA of TRPC1 expression

The TRPC1 transcript levels were measured by real-time PCR. The PCR primers for TRPC1 were designed as follows: TRPC1 forward, 5′-GCCAGTTTTGTCACTTTGTTATTT-3′; TRPC1 reverse, 5′-CCCATTGTGTTTTTCTTATCCTCA-3′; GAPDH forward, 5′-CGCTGAGTACGTCGTGGAGTC-3′; and GAPDH reverse, 5′-GCTGATGATCTTGAGGCTGTTGTC-3′ (TaKaRa Biotechnology, Japan).

The pulmonary tissues were maintained in formalin, routinely processed, and embedded in paraffin. TRPC1 expression was assessed according to immunohistochemical methods [33]. The rabbit anti-TRPC1 polyclonal antibody was diluted 1:25.

Frozen bronchial tissue was homogenized in 200 mL of RIPA lysis buffer containing protease inhibitors. This mixture was then centrifuged. The supernatants of *A* group were used for ELISA (ELISA kit (R&D systems, USA)) and the supernatants of of *B* group were used for western blot analysis (1:1000; Abcam, USA) [26], GAPDH was used by 1:1000 (Beyotime, China).

### Cytokines in bronchial BALF and 16HBE cells

The TGF-β_1_ protein in 16HBE cells was measured by western blot(1:1000; Abcam, USA), GAPDH was used by 1:1000 (Beyotime, China) [26]. The secreted IL-13, MMP-9 and TGF-β_1_ in BALF were measured using ELISA kit (R&D systems, USA).

### Statistical

The data are reported as the means ± SD. The data analyses were performed with SPSS software v. 10.0. In the analyses, paired-*t* tests were used for the comparisons between two groups. *P <* 0.05 were considered statistically significant.

## Results

### TRPC1 siRNA knockdowned TRPC1 expression successfully in bronchial epithelial cell group

**Fig. 1:**
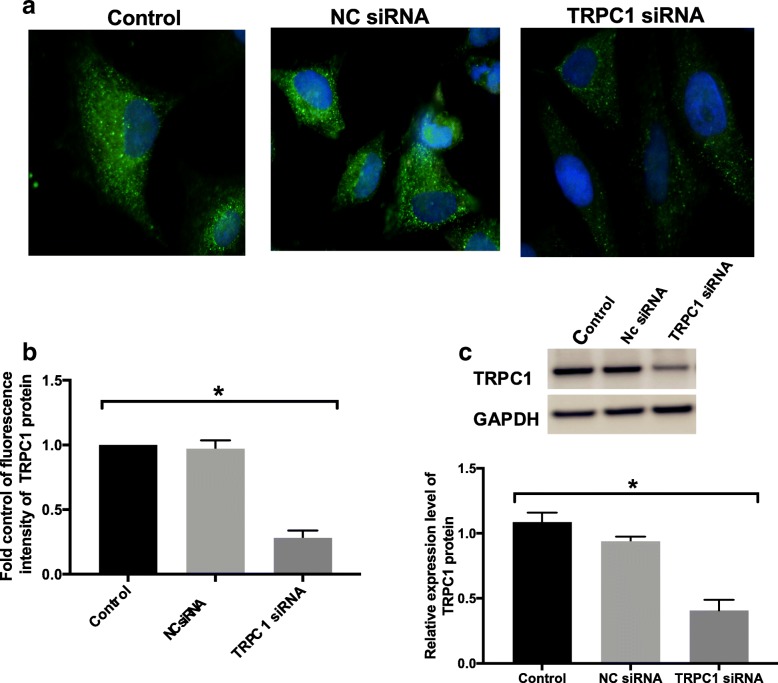
The successful transfection and knockdown efficiency of TRPC1 siRNA. **a-c** The. knockdown efficiency of TRPC1 siRNA in TRPC1 expression was determined using. Immunofluorescence (X400) and western blot analysis. **b** and **c** Fold control of. immunofluorescence values and relative protein expression level of TRPC1 are shown as the. Means ± SD (*n* = 3). *P < 0.05

The efficiency of knockdown was detected using immunofluorescence and western blot. A significantly lower level of TRPC1 protein was observed in the 16HBE cells stably transfected with TRPC1 siRNA than control group (Fig. 1). However, there was no difference between NC siRNA and control group (Fig. 1b and c).

### Activation of TRPC1 channels in 16HBE cells treated with mechanical stretch

**Fig. 2:**
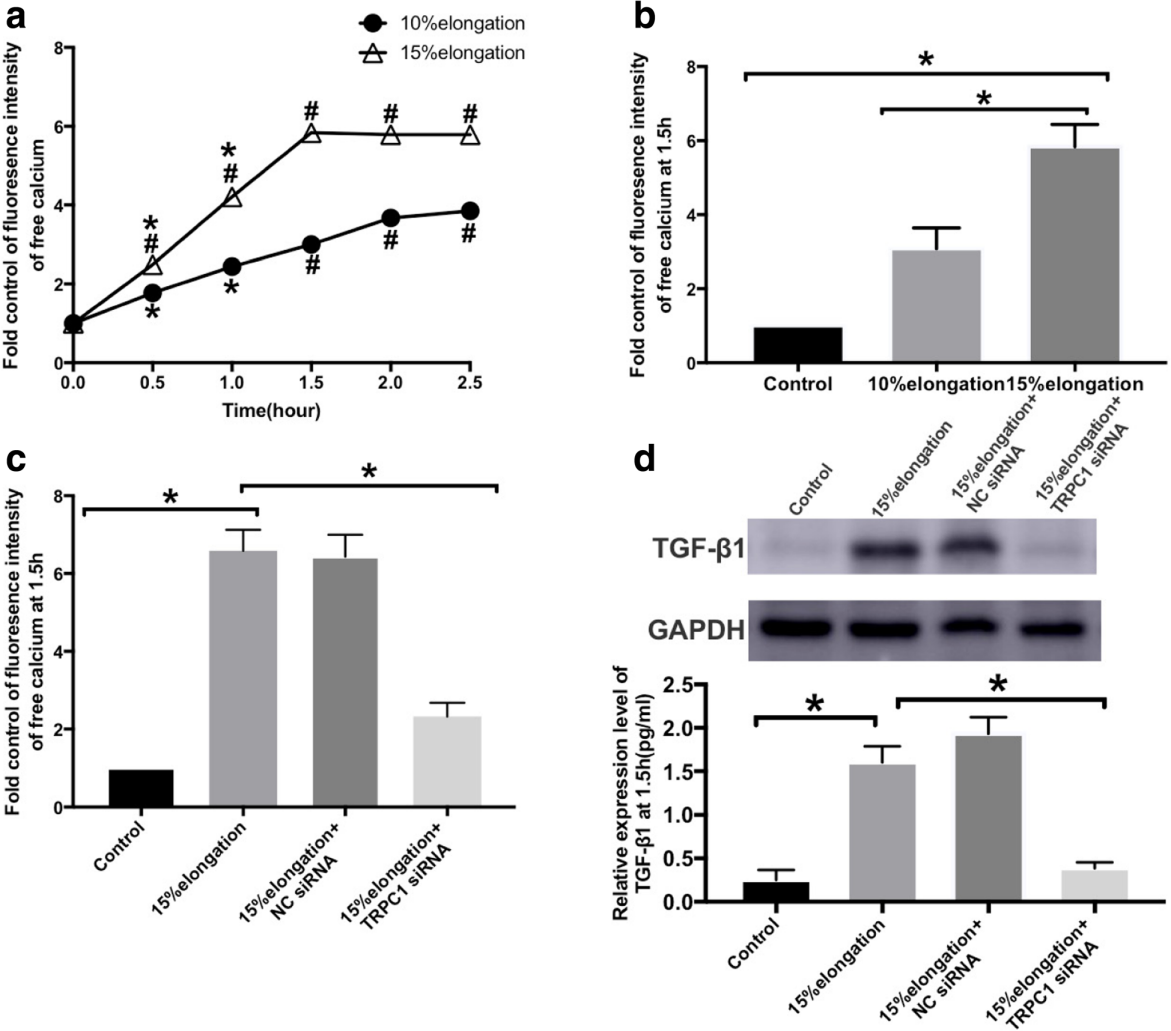
The level of Ca2+ fluorescence intensity after mechanical stretch was observed using. Laser confocal microscopy. The fluorescence intensity was shown by fold control. **a** 16HBE. were cells stretched for 0, 0.5, 1, 1.5, 2, and 2.5 h at 10 and 15%. #*P* < 0.05 versus. Control group (cells stretched for 0 min); and *P < 0.05 versus cells stretched for 1.5 h. **b** Cells were stretched for 1.5 h at different levels of elongation. *P < 0.05. **c** The effect of. TRPC1 siRNA on intracellular Ca2+ flux under 15% elongation for 1.5 h. * *P* < 0.0*5*. In stretch.v group, 15% elongation for 1.5 h was chosed for further experiment. **d** Expression level of TGF-. β1 was measured at 15% elongation for 1.5 h by western blot. Values are means ± SD (*n* = 3). *. *P* < 0.0*5*

We used Ca^2+^ imaging to explore the activation of TRPC1 in 16HBE cells stimulated by cyclic stretch. The maximum Ca^2+^ flux was achieved after cells were exposed to stretch for 1.5 h and it appeared to plateau from 1.5 h to 2.5 h in the 10 and 15% elongation groups (Fig. 2a). At 15% elongation, the increase in intracellular Ca^2+^ was significantly higher than 10% elongation (Fig. 2a and b). Therefore, 15% elongation was chosen for further experiments, and 1.5 h as the best time to evaluate intracellular Ca^2+^ levels. The stretch-induced Ca^2+^ was markedly attenuated in the 16HBE cells that were transfected with TRPC1 siRNA but not in the 16HBE cells transfected with NC siRNA (Fig. 2c). These results indicate that TRPC1 plays a vital role in stress-induced Ca^2+^ flux in 16HBE cells.

### Effect of TRPC1 siRNA treatments on IL-13, MMP-9 and TGF-β_1_ protein expression levels in the 1.5 h mechanical stretch group

Previous studies [26] showed that the peak protein expression levels of IL-13 and MMP-9 were observed after exposure to mechanical pressure in vivo. TRPC1 siRNA attenuated IL-13 and MMP-9 protein expression. In this study, TRPC1 siRNA reduced the expression level of TGF-β_1_ in 16HBE. TGF-β_1_ protein level in the 15% elongation +TRPC1 siRNA group were significantly lower than those in 15% elongation group (Fig. 2d).

### Depleted TRPC1 expression by TRPC1 siRNA successfully using a MicroSprayer aerosolizer

**Fig. 3:**
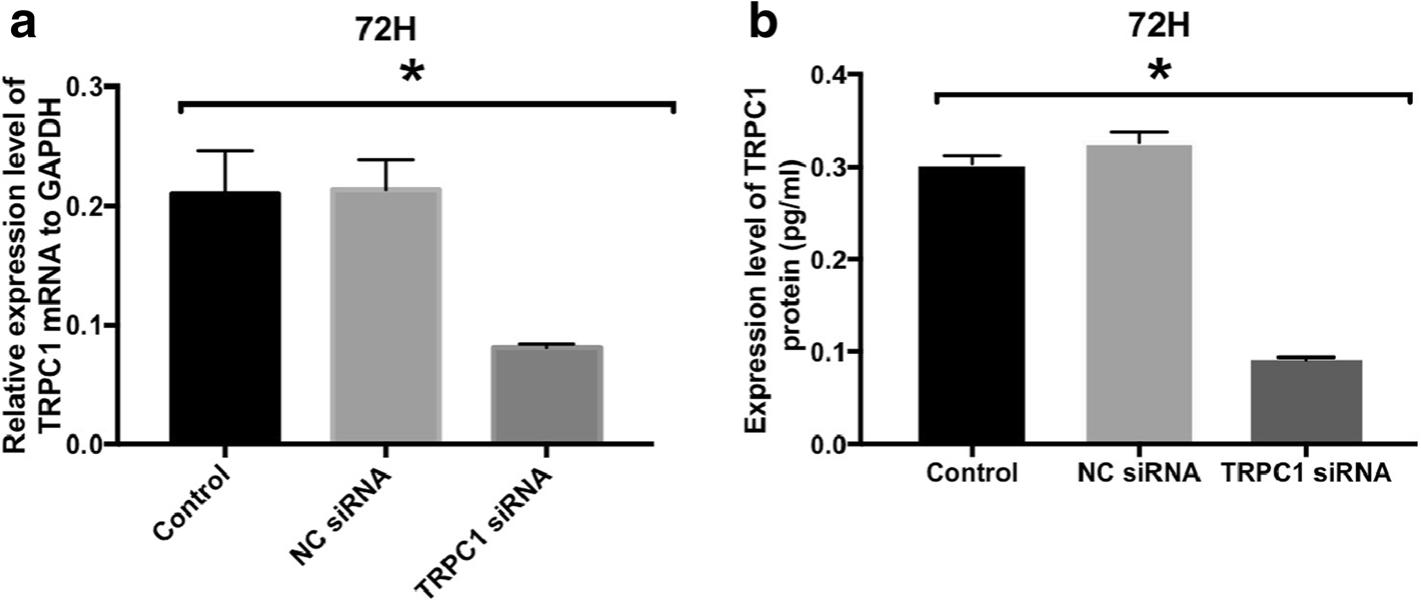
Intervention effect of TRPC1 siRNA on mRNA and protein expression after 72 h. **a** The. mRNA expression of TRPC1 was analyzed by real-time PCR relative to GAPDH. **b** The. expression level of TRPC1 protein was measured by ELISA. Values are means ± SD (*n* = 3). *P. < 0.05

The changes in TRPC1 mRNA and protein expression in the *A* group animals after siRNA exposure were further analysed by real-time PCR and ELISA, respectively. Seventy-two hours later, compared with control group, expression level of TRPC1 mRNA and protein in TRPC1 siRNA group decreased (Fig. 3a and b). Results demonstrated the successful transfection of TRPC1-targeted siRNA in bronchial airway tissue.

### Validation of asthmatic models

**Fig. 4:**
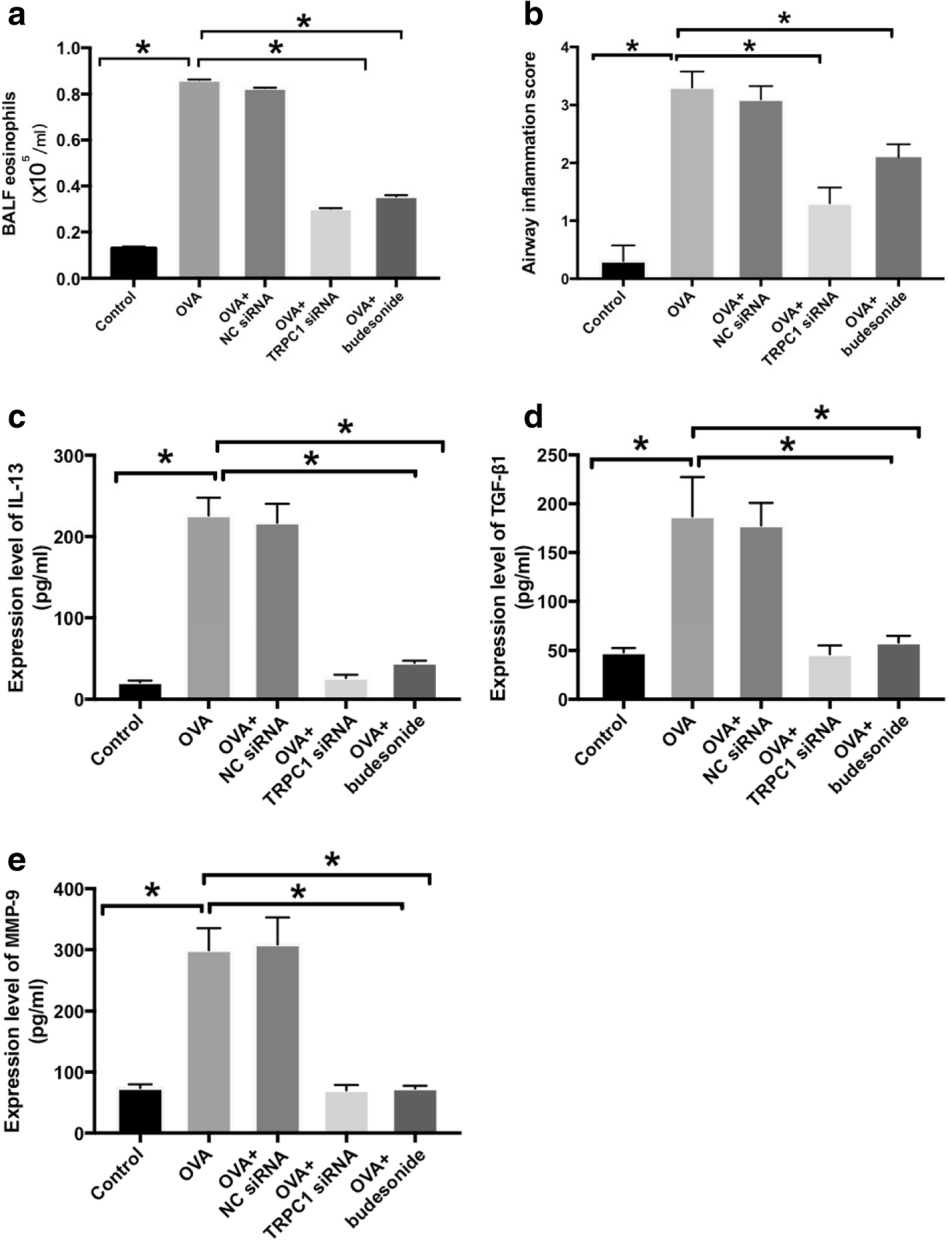
Animals were administered ovalbumin and aluminum hydroxide for establishing. Asthmatic models. The influence of TPRC1 knockout on airway inflammation also could be. seen above. **a** The number of eosinophils in the BALF was assessed by Wright Giemsa. staining. **b** The inflammatory cell infiltration around the airway was scored by HE staining. **c** IL-13 was assayed using an ELISA in BALF. **d** TGF-β1 protein was measured by ELISA in. BALF. **e** MMP-9 protein was measured by ELISA in BALF. The down-regulated expression of. TRPC1 exerts significant influence on airway inflammation infiltration. Values are means ± SD. (*n* = 5). OVA: ovalbumin. *P < 0.05

**Fig. 5:**
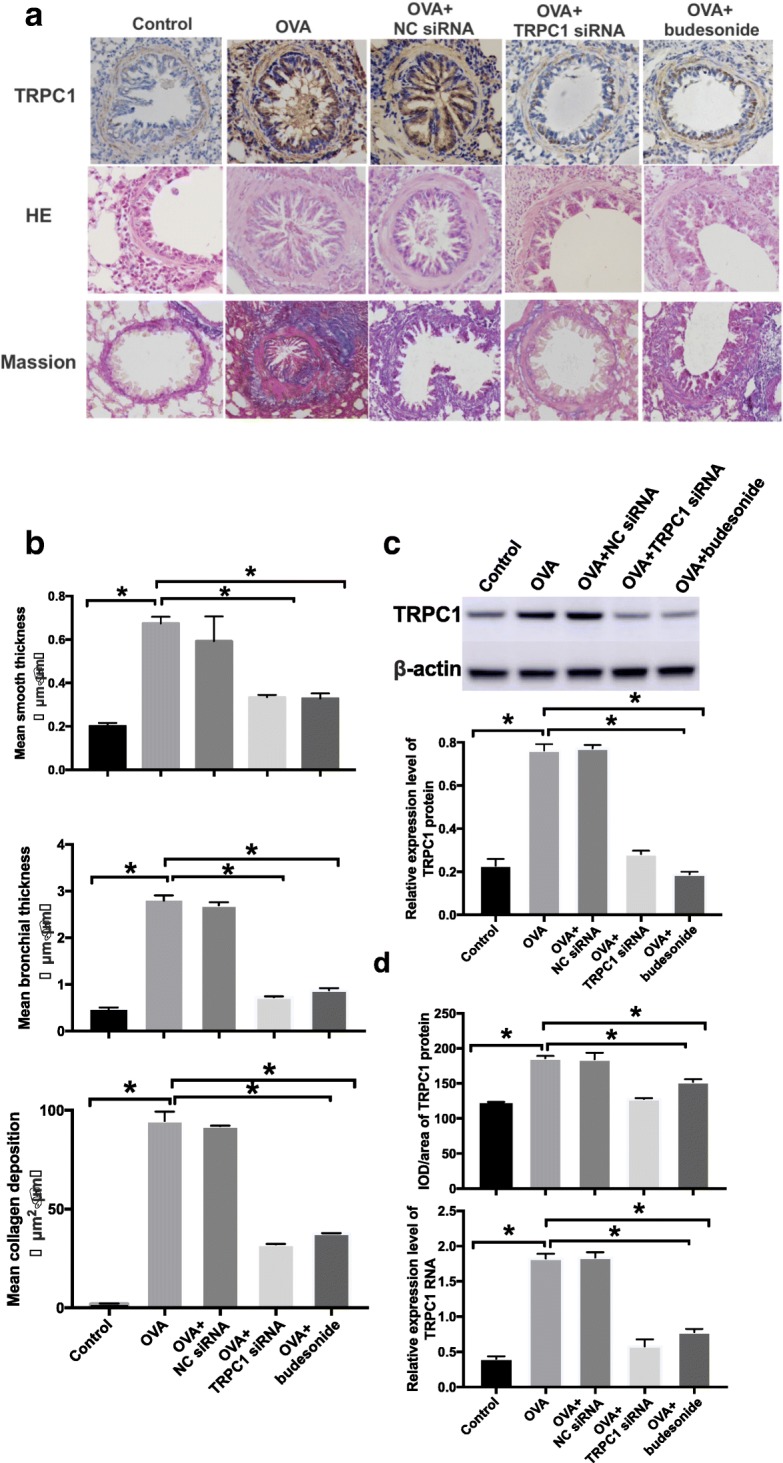
Analysis of TRPC1 expression and airway remodeling for the control, OVA,. OVA+TRPC1 siRNA, OVA+NC siRNA and OVA+budesonide groups. **a** Pulmonary sections. Were subjected to immunohistochemistry experiments, HE and Masson staining. All. photomicrographs were captured at × 400. **b** We observed different airway wall thicknesses,. smooth muscle thicknesses and collagen deposition area. **c** Quantification of TRPC1 protein. Was performed by western blot. β-actin served as the internal control. **d** IOD/area of TRPC1. protein and expression of TRPC1 mRNA are shown above. The results above mean that. TRPC1 could active and accelerate the airway remodeling. All data were collected after eight. Weeks sand shown as the means ± SD (*n* = 5). OVA: ovalbumin. *P < 0.05

Ovalbumin exposure can be used to successfully construct asthma models [27]. The number of BALF eosinophils in the OVA group was significantly higher than that obtained in the control group (Fig. 4a). The score of inflammatory cells (such as eosinophils mainly) in pulmonary parenchyma was elevated in OVA group compared with control group (Fig. 4b). The thickness of bronchial wall, smooth muscle and ECM deposition area were increased in ovalbumin-treated group compared with control group (Fig. 5a and b). Results suggest that airway remodeling were successfully established in the chronic allergic airway disease models.

### Effect of ovalbumin exposure on TRPC1 mRNA and protein

The immunohistochemical experiments showed that TRPC1 protein was significantly upregulated in the bronchial epithelium layer in the asthmatic animals, with a majority of proteins located in the columnar epithelium, goblet cells and basal membrane and a minority distributed among the bronchial smooth muscle and matrix (Fig. 5a). Additionally, we observed that the TRPC1 protein levels in the OVA group were markedly higher than those of the control group (Fig. 5c). The integrated optical density (IOD) divided by area of TRPC1 proteins in the bronchial epithelium layer was increased significantly in asthmatic animals compared with the control group (Fig. 5d). A real-time PCR analysis demonstrated that TRPC1 mRNA expression was increased significantly in the OVA group compared with the control group (Fig. 5d).

### TRPC1 weakened the airway inflammation and airway remodeling of asthmatic animals, showing similar treatment effect with budesonide

In OVA+TRPC1 siRNA group and OVA+budesonide group, eosinophils in BALF were significantly lower than that of OVA group (Fig. 4a). The peak level of inflammatory cells infiltration scores, protein levels of IL-13, MMP-9 and TGF-β1 in OVA+TRPC1 siRNA group and OVA+ budesonide was significantly reduced than those in the OVA group (Fig. 4b-e). Results proved that the anti-inflammatory therapeutic treatment of TRPC1 siRNA was similar with budesonide. There was no significant difference between OVA+NC siRNA group and OVA group (Fig. 4).

The histopathological experiments revealed varying degrees of airway remodeling in the OVA, OVA+TRPC1 siRNA, OVA+NC siRNA and OVA+budesonide groups. In detail, the OVA and OVA+NC siRNA group exhibited the most notable changes in airway remodeling (Fig. 5a and b). However, the silencing of TRPC1 with siRNA decreased the bronchial wall thickness, abnormal smooth muscle thickness and subepithelial matrix deposition (Figs. 5a and b).

The peak level of bronchial wall thickness, abnormal smooth muscle thickness and subepithelial matrix deposition in OVA+budesonide showed lower than OVA group (Fig. 5a and b). The IOD/area of the TRPC1 protein in the OVA+budesonide group was also decreased (Fig. 5a and d). The western blot results proved to be same as those described above (Fig. 5c). The TRPC1 mRNA expression level in the OVA+budesonide group was decreased significantly compared with that of the OVA group (Fig. 5d). This result suggests that budesonide could alleviate the process of remodelling, however the relationship with TRPC1 still remain unclear. The TRPC1 mRNA and protein levels observed in the OVA+NC siRNA group were similar compared with those of the OVA group (Fig. 5a, c and d).

## Discussion

Airway constriction and cough can impose compressive stress on the airway epithelium during attack of asthma [2–7]. Mechanosensitive cation channel (MscCa) are universally found in mammal cell membranes, which could be gated by olfaction, mechanical, chemical, temperature, pH, osmolarity and vasorelaxation of blood vessels [18]. Studies have shown that mammal MscCas are encoded by TRP gene [18, 25]. Hence, TRPC1 is recognized as a mechanical pressure-sensitive calcium ion channel.

To investigate the mechanical sensitivity of TRPC1 in epithelial cells [35], the optimal activity of TRPC1 was detected at 1.5 h, 15% elongation by Ca^2+^ imaging [26]. Because pulmonary epithelial cell stretch is accompanied by increased mechanical stress in airways, cyclic stretch was used to imitate airway mechanical stress by Flex cell Tension System. Related studies showed that 18% elongation was widely applied to human pulmonary artery endothelial cells and pulmonary microvascular endothelial cells in ventilator-induced pulmonary injury [36–38]. 12% elongation was applied in airway smooth muscle hypertrophy using Flex cell Tension System [39]. Studies also proved that inducing cyclic stretch at 15% elongation corresponds to an 12 to 20% linear distension in the epithelium [23, 24]. Thus, the peak free intracellular calcium was observed at 15% elongation for 1.5 h in 16HBE. Under mechanical stimuli, the peak expression level of free calcium were inhibited by TRPC1 siRNA. Thus we determine that Ca^2+^ invoked by TRPC1 channel mediates mechanotransduction mechanism in bronchial epithelial cells.

In vivo, higher peak protein and mRNA expression level of TRPC1 were found in OVA group than control group. Most proteins were localized in the epithelium layer along the inside of the bronchial wall. Also, small amount of TRPC1 was displayed among bronchial smooth muscle cells in vivo, which was consistent with previous reports [40–42]. After the successful construction of asthmatic models, pulmonary tissue biopsy using HE and Massion straining revealed marked abnormal structural changes, such as increased airway wall thicknesses, abnormal collagen deposition below sub-epithelial layer, smooth muscle hyperplasia and hypertrophy. Due to the absence of specific TRPC1 blockers, siRNA solution was applied using a MicroSprayer aerosolizer [28]. However, TRPC1 siRNA could inhibit the expression level of TRPC1 and significantly impaired airway remodeling in asthmatic models. Similarly, the application of budesonide also showed similar expression level of TRPC1 and impaired airway remodeling. It proved that TRPC1 plays an important role in airway remodeling, which represents significant clinic therapeutic effect in asthma.

Combined with our *vivo* and *vitro* studies, mechanical stress could act directly on bronchial epithelial cells, which is related with the molecular structure of an antenna-like sensor TRPC1 channel [14, 18]. TRPC1 channel was determined as the strongest candidate component of store-operated calcium entry [19]. Under mechanical stimuli, TRPC1 primarily functions as a cation non-selective channel within pathways controlling calcium entry, affecting basic cell functions, such as proliferation and survival, differentiation, secretion, and cell migration [14].

Ca^2+^ signalling has been implicated in the induction of TGF-β_1_ expression in a model of fibrosis [43]. Active TGF-β_1_ is also found in the BALF taken from subjects with asthma [54]. TGF-β_1_ stimulates the recruitment of inflammatory cells to the airway and promotes the secretion of additional growth factors and inflammatory cytokines [43]. After airway injury, TGF-β_1_ promotes the differentiation of epithelial cells, fibroblasts and airway smooth muscle (ASM) cells into a more contractile phenotype [44]. In addition, TGF-β_1_ induces greater proliferation in human ASM cells from patients with severe asthma as compared with those from patients with nonsevere asthma [45].

Another remodeling mediator also has been identified--MMP-9. Study showed that increased level of MMP-9 also relies on a calcium signaling pathway [46]. In OVA-induced chronic allergic airway disease, increased MMP-9 level in BALF also could be found [47], which plays an important role in the tissue remodeling associated with various pathological processes such as morphogenesis, angiogenesis, tissue repair, migration and metastasis [48, 49]. And MMP-9 is produced by epithelial and inflammatory cells and up-regulated release of matrix-associated TGF-β_1_, which promote pathological airway remodeling in patients with asthma [50].

Previous reports have suggested that TRPC1-coupled Ca^2+^ is a potential proinflammatory factor in allergen-induced pulmonary disease, which may result in airway hyperreaction (AHR) in asthmatic patients [51]. IL-13 is a Th2 cytokine, participates in the regulation of airway inflammation, which induces airway remodeling [52]. IL-13 is involved in mucus production and AHR [53]. IL-13 also contributes to epithelial cell maturation and bronchial fibrosisin in asthma [53, 54].

Combined with our previous results [26], we analyzed the expression levels of TGF-β_1_, MMP-9 and IL-13 in vivo and *vitro*. The numbers of eosinophils in BALF and inflammation score arround airway were calculated. Under mechanical stimuli or asthamtic condition, the protein level of TGF-β_1_, MMP-9 and IL-13 showed higher than control group. Once using the TRPC1 siRNA or budesonide, the expression level of TGF-β_1_, MMP-9, IL-13, eosinophils in BALF and inflammation score reduced. In additional, a novelty muti-scale computational Q3D method will further be used for the quantitation of inflammation [55]. Budesonide can decrease the relevant changes during airway remodeling, companied with decreased TRPC1. However, the mechanism between TRPC1 and budesonide still remain unclear.

## Conclusion

Although the mechanism through which TRPC1 regulates the described events is yet to be determined, TRPC1-coupled Ca^2+^ signalling plays an important role in airway remodeling. Ultimately, these events constitute disorders of the complex airway and bronchial tissues and further lead to airway remodeling, which aggravates the airway hyperresponsiveness and fixed airway obstruction. The development of drugs that target the function and expression of TRPC1 may be an additional strategy for developing novel therapeutic approaches for asthma that prevent progressive airway remodeling.

## Abbreviations

AHR: Airway hyperreaction
AJE: American Journal Experts
ASM: Airway smooth muscle
BALF: Bronchialveolar liquid
ECM: Intracellular and extracellular matrix
ELISA: Enzyme linked immunosorbent assay
HBE: Human bronchial epithelial cells
HE: Haematoxylin-eosin
IL-13: Interleukin-13
IOD: Ratio of integrated optical density
MMP: Matrix metalloproteinase
MscCa: Mechanosensitive cation channel
MUC5AC: Mucin 5 AC
OVA: Ovalbumin
PCR: Polymerase chain reaction
TGF: Transforming growth
TRPC1: Transient receptor potential channel 1
